# One‐Stage Arthroscopic Multiple Ligament Reconstruction for Schenck IV Knee Dislocation

**DOI:** 10.1111/os.13611

**Published:** 2022-12-13

**Authors:** Chao Li, Yubo Liu, Runlong Zheng, Jitong Sun, Wei Peng, Xiang‐Hua Deng, Xunwu Huang

**Affiliations:** ^1^ Senior Department of Orthopaedics The Fourth Medical Center of PLA General Hospital Beijing China; ^2^ National Clinical Research Center for Orthopaedics Sports Medicine & Rehabilitation Beijing China; ^3^ School of Medicine Nankai University Tianjin China; ^4^ Department of Orthopaedic Surgery PLA Strategic Support Force Characteristic Medical Center Beijing China; ^5^ Sports Medicine and Shoulder Service Hospital for Special Surgery New York USA; ^6^ Orthopaedic Soft Tissue Research Program Hospital for Special Surgery New York USA

**Keywords:** Knee Function, Ligament Reconstruction, One‐Stage Arthroscopic Surgery, Schenck IV Knee Dislocation, Surgical Approach

## Abstract

**Purpose:**

Schenck IV knee dislocation patients have dissatisfactory knee function and return‐to‐sport rate with the existing treatment methods. The purpose of this study was to illustrate a one‐stage arthroscopic multiple ligament reconstruction method for treating Schenck IV knee dislocations.

**Methods:**

A retrospective case series study was performed. All patients with a history of Schenck IV knee dislocation who underwent one‐stage arthroscopic multi‐ligament reconstruction from 2010 to 2018 were followed for 24 months. The outcomes, including general patient data, Lysholm scores, International Knee Documentation Committee (IKDC) scores, visual analog scale (VAS) pain scores, knee active range of motion, and complications, were reviewed. The data was analyzed with paired‐samples t‐test.

**Results:**

A total of 12 patients, comprising nine males and three females, were followed up and reviewed. The mean age at the time of the surgical procedure was 40.3 ± 9.0 (22–57) years. The mean body mass index (BMI) was 24.6 ± 4.9 (15.2–32.5) kg/m^2^. The mean IKDC score and Lysholm score before surgery were 30.4 ± 6.1 (21–42) and 28.2 ± 6.2 (22–39), respectively. The average operation time was 121.8 minutes. The mean IKDC score and Lysholm score at the 24‐month follow‐up were 80.6 ± 6.5 (68–92) and 82.0 ± 7.5 (72–95), respectively. There were significant differences in the IKDC and Lysholm scores between the preoperative and 24‐month postoperative time points (*p* < 0.01). The mean knee range of motion was 124.6° ± 6.6° (115°–135°) at the 24‐month follow‐up. No major complications occurred.

**Conclusions:**

The results of this retrospective study suggest that the new arthroscopic one‐stage multi‐ligament reconstruction technique is an effective way to treat Schenck IV knee dislocation with satisfactory postoperative knee function.

## Graphical abstract

The ligament reconstructions were performed following the order of PCL, MCL, ACL, and LCL. The waved end of one ligament was pulled with a guide wire following the order of points 1‐2‐4‐3‐10, and the guide wire was pulled through tunnel E and out of point 9. Another waved end of the ligament was pulled with a guide wire following the order of points 5‐6‐8‐7‐9, and the guide wire was pulled through tunnel E and out of point 10. The ligaments were pulled tightly, and interference screws were placed at points 3, 7, 9, and 10. Sometimes the bony end of the tendon at point 1 or point 5 may be waved and reinforced by an interface screw if the bone–tendon interface is dehiscent.

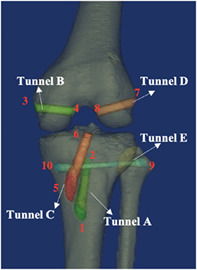

## Introduction

Traumatic knee dislocation (TKD) is mostly caused by high‐energy trauma, leading to serious damage to the tissues around the knee. It used to be a rare injury accounting for 0.02%–0.2% of all orthopaedic injuries.^1^, ^2^ However, the incidence might have been underestimated because of misdiagnoses and spontaneous knee reduction.^3^ Additionally, morbidity may rise with the increase in high‐energy trauma, low‐velocity injuries (contact sports), or ultralow‐velocity injuries among obese populations.^4^, ^5^, ^6^ Thus, considering the large population base, the patient number is substantial, especially in developed regions where the prevalence of obesity and high‐energy trauma is higher than that in developing regions.

The classification system proposed by Schenck et al.^7^ in 1994 and modified by nerve and vascular injury subtypes by others has been commonly used today in treating knee dislocation. This classification system is based on anatomically injured structures and is more valuable in making surgical plans.

Existing studies show that patients diagnosed with Schenck IV and V knee dislocation (KD) have exhibited poorer knee functions and return to sport rates than patients with other subtypes. Everhart et al.^8^ reported a systematic review of 21 studies on 524 patients and found that patients with Schenck IV or V KD injuries have lower return to work rate compared with other KD subtypes Werner et al.^9^ reviewed 65 patients with knee dislocation and followed them for 12 years and found significantly better knee function for KD III‐M patients than for KD IV patients. This conclusion was also supported by several other stydies.^10^, ^11^ Considering the unsatisfactory prognosis, it is imperative for us to find and illustrate some new treatment methods to improve the knee functions of Schenck IV patients.

To our knowledge, most studies to date have considered all KD subtypes together; very few studies have focused specifically on the treatment of Schenck IV KD. The purpose of this study was to: (i) illustrate a surgical method that we developed; (ii) describe the operative characteristics and follow‐up outcomes of this method in treating Schenck IV KD patients; (iii) discuss the advantages of the surgical method. Thus, this study aimed to provide a new option for the treatment of Schenck IV KD patients.

## Materials and Methods

### Patients' Data

Patients who met the following inclusion criteria were enrolled in the research: (i) patients in our database who suffered from Schenck IV KD; (ii) underwent arthroscopic one‐stage multi‐ligament reconstruction surgery; (iii) from January 2010 to January 2018. Patients who met the following criteria were excluded from the research: (i) over 70 years old; (ii) had artery injuries; (iii) nerve injuries; (iv) compartment syndrome; (v) proximal tibiofibular separation. All surgeries were performed by one senior surgeon. Patient data, including age, sex, body mass index (BMI), cause of injury, waiting time before surgery, and operation time, were collected. IKDC score, Lysholm score, VAS score, postoperative knee range of motion and complications were reviewed at timepoints of 3 months, 6 months, 12 months, 24 months post operation and analyzed with paired‐samples t‐test. α = 0.05 was set as the risk level and *p* < 0 .05 was considered to be statistically significant.

The study protocol was approved by the IRB of authors' affiliated institution (No. 8210090128), and all the participating patients were fully informed and participated in the study voluntarily.

### Surgical Technique

All patients were promptly repositioned after admission and then protected with a plaster cast. A thorough physical examination, knee magnetic resonance imaging, CT angiography, and blood vessel ultrasound were performed. Operations were performed under a combination of spinal epidural anesthesia and general anesthesia. The knee was kept stable during the preoperative preparation to avoid neurovascular damage. The standard arthroscopic approach was used to clean out the intra‐articular hemorrhage, residual ligament tissue, protruding joint capsule, and synovial tissue. Patients with meniscal injuries were given meniscus sutures or partial meniscus resection depending on the type and size of the injury. Patients with cartilage injury were treated with cartilage surface cleansing or microfracture. Deep‐frozen homologous Achilles tendons were thawed in normal saline with gentamicin for 20 min and then cut into two even parts along the grain, with the calcaneus still attached. The tendon was trimmed down to 8 mm in diameter through the distal 2/3 and 5 to 6 mm through the proximal 1/3, and a 10 mm piece of bone was left attached. The proximal 2/3 of the tendon was weaved and sutured with non‐absorbable sutures (W4843, Ethibond Excel, Johnson & Johnson).

**Fig. 1:**
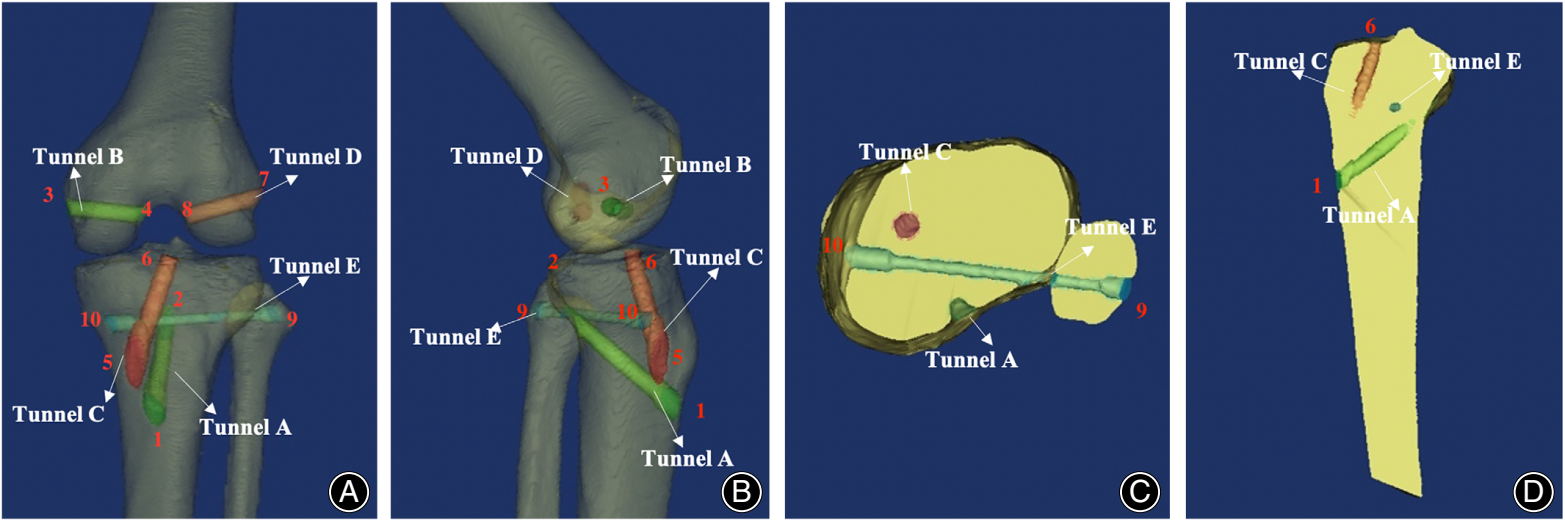
Illustration of the reconstruction model. Figure shows the bone tunnels of the new multiligament reconstruction technique. The three‐dimensional knee model was constructed by MIMICS (Materialize's interactive medical image control system) using data from the CT scan of a healthy volunteer. The bone tunnels were built with MIMICS according to the methods used in the surgical procedure. (A, B) Shows the frontal and lateral views of the reconstructed model. (C, D) Shows the positions of the tibial bone tunnels

Ligament reconstructions: Bone tunnels were created as follows. Tunnel A: The guide tip was placed 15 mm below the joint line in the posterior cruciate ligament (PCL) facet (point 2). The drill guide was oriented approximately 60° from the articular surface of the tibia, starting 5 mm medial to the tibial anterior ridge (point 1). A tibial tunnel with a diameter of 8 mm was drilled from point 1 to point 2 for the reconstruction of the PCL, and the proximal end was expanded to 10 mm to accommodate the bone attached to the tendon. Tunnel B: A femur bone tunnel with a diameter of 8 mm was drilled from the attachment point of the medial collateral ligament, or MCL (point 3), on the medical femoral epicondyle to the anterior border of the PCL anterolateral bundle footprint (point 4). Tunnel C: The guide tip was placed 1.5 mm ahead of the center of the tibial footprint of the anterior cruciate ligament, or ACL (point 6); the drill guide was oriented approximately 55°, starting 2 cm medial to the tibial anterior ridge (point 5); a tibial bone tunnel with a diameter of 8 mm was drilled from point 5 to point 6 for the reconstruction of the ACL; and the proximal end was expanded to 10 mm to accommodate the bone attached to the tendon. Tunnel D: A femur bone tunnel with a diameter of 8 mm was drilled from the attachment point of the lateral collateral ligament (LCL) on the femur (point 7) to the posterior superior border of the ACL anteromedial bundle footprint (point 8). Tunnel E: A 4 mm tunnel was drilled from the midpoint of the widest part of the fibular head (point 9) to the upper border of the distal tibial attachment of the superficial layer of the MCL (point 10). The two ends of this tunnel were expanded to 8 mm for the tendon and interference screws. The bone tunnels are shown in Figure 1.The ligament reconstructions were performed in the following order: PCL, MCL, ACL, and LCL. The waved end of one ligament was pulled with a guide wire to points 1, 2, 4, 3, and 10 in that order, and the guide wire was pulled through tunnel E and out of point 9. The waved end of another ligament was pulled with a guide wire to points 5, 6, 8, 7, and 9 in that order, and the guide wire was pulled through tunnel E and out of point 10. The ligaments were pulled tightly, and interference screws were placed at points 3, 7, 9, and 10. Sometimes the bony end of the tendon at point 1 or point 5 may be waved and reinforced by an interface screw if the bone–tendon interface is dehiscent.

### Postoperative Management

The knee joint was kept at 0° using a brace with a chuck brace for 3 weeks after surgery, and the patient was encouraged to walk and exercise the quadriceps while wearing the brace after 3 weeks. The angle of the chuck brace was limited to a range of 0°–30° in the 4th week, 0°–60° in the 5th week, 0°–90° in the 6th week, 0°–120° in the 7th week, and 0°–135° in the 8th week. From the 9th week to the 15th week, the patient was encouraged to perform daily exercise with the protection of the chuck brace with no angle limitations but were still instructed to avoid weight‐bearing exercise.

## Results

### General Results

A cohort of 12 patients was reviewed. The patients comprised nine males and three females. The mean age was 40.3 ± 9.0 (22–57) years. The mean BMI was 24.6 ± 4.9 (15.2–32.5) kg/m^2^. The mean waiting time for surgery ranged from 4 to 42 days. The average operation time was 121.8 minutes. We observed one traumatic splenic rupture in one patient, combined bone fractures in three patients (one tibia, one fibula, one rib), one fibular fracture in one patient, and deep vein thrombosis in two patients. Meniscal injury was observed in three patients, and articular cartilage injuries were observed in three patients.

### Clinical Improvement

The mean IKDC scores before surgery and at 3 months, 6 months, 12 months, and 24 months postoperatively were 30.4 ± 6.1, 35.3 ± 4.0, 50.4 ± 5.9, 68.4 ± 6.4, and 80.6 ± 6.5, respectively. The mean Lysholm scores before surgery and at 3 months, 6 months, 12 months, and 24 months postoperatively were 28.2 ± 6.2, 45.8 ± 5.6, 69.9 ± 6.3, 76.8 ± 6.0, and 82.0 ± 7.5, respectively. There were significant differences in the IKDC score and Lysholm score between the preoperative timepoint and the 24‐month postoperative follow‐up (*p* < 0.01). The patients who underwent surgery within 3 weeks of injury had a higher IKDC score (83.8 ± 6.0) than the patients who underwent surgery more than 3 weeks after injury (76.3 ± 4.2). The VAS scores before surgery and at the 3rd, 6th, 12^th^, and 24th months postoperatively were 6.8 ± 1.3, 4.6 ± 1.4, 3.4 ± 1.4, 1.8 ± 0.9, and 1.3 ± 0.9, respectively. The mean active range of motion of the knee at the 3rd month, 6th month, 12th month, and 24th month postoperatively measured 105.8° ± 7.6°, 117.5° ± 5.0°, 124.6° ± 6.6°, and 124.6° ± 6.6°, respectively.

### Complications

One patient received arthroscopic arthrolysis because of postoperative joint adhesions. One patient had popliteal vein thrombosis and recovered after anti‐coagulation therapy. No other major complications occurred. The patients' general data are shown in Table 1, and the IKDC scores, Lysholm scores, VAS scores, and postoperative active range of motion are shown in Table 2.

**TABLE 1:** Patient characteristics

Characteristics	Values
Age (years)	40.3 ± 9.0 (22–57)
Gender (male: female)	9:3
BMI (kg/m^2^)	24.6 ± 4.9 (15.2–32.5)
Waiting time (days)	4–42
Operation time (minutes)	121.8
Follow up time (months)	24

**TABLE 2:** Patient follow‐up data

Time	Before operation	3 months	6 months	12 months	24 months
IKDC score	30.4 ± 6.1	35.3 ± 4.0	50.4 ± 5.9	68.4 ± 6.4	80.6 ± 6.5
Lysholm score	28.2 ± 6.2	45.8 ± 5.6	69.9 ± 6.3	76.8 ± 6.0	82.0 ± 7.5
VAS score	6.8 ± 1.3	4.6 ± 1.4	3.4 ± 1.4	1.8 ± 0.9	1.3 ± 0.9
Knee active range of motion (°)	‐	105.8 ± 7.6	117.5 ± 5.0	124.6 ± 6.6	124.6 ± 6.6

### Typical Case

**Fig. 2:**
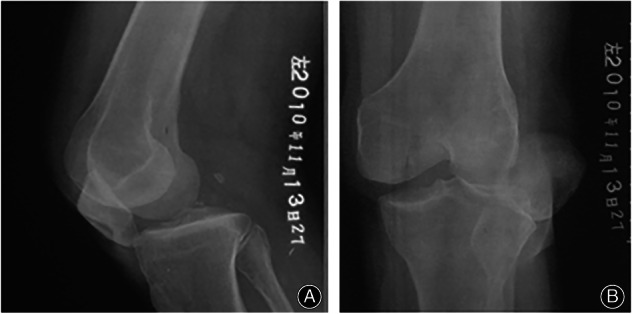
A 48‐year‐old male underwent arthroscopic multiple ligament reconstruction, partial meniscectomy and debridement for Schenck IV dislocation of the left knee. (A, B) X‐ray shows the dislocated knee

**Fig. 3:**
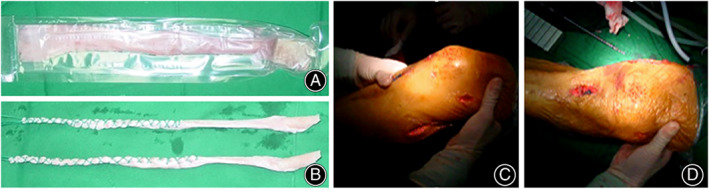
Intraoperative conditions. (A, B) A deep‐frozen homologous Achilles tendon before and after preparation. (C, D) Intraoperative pictures show the incisions

**Fig. 4:**
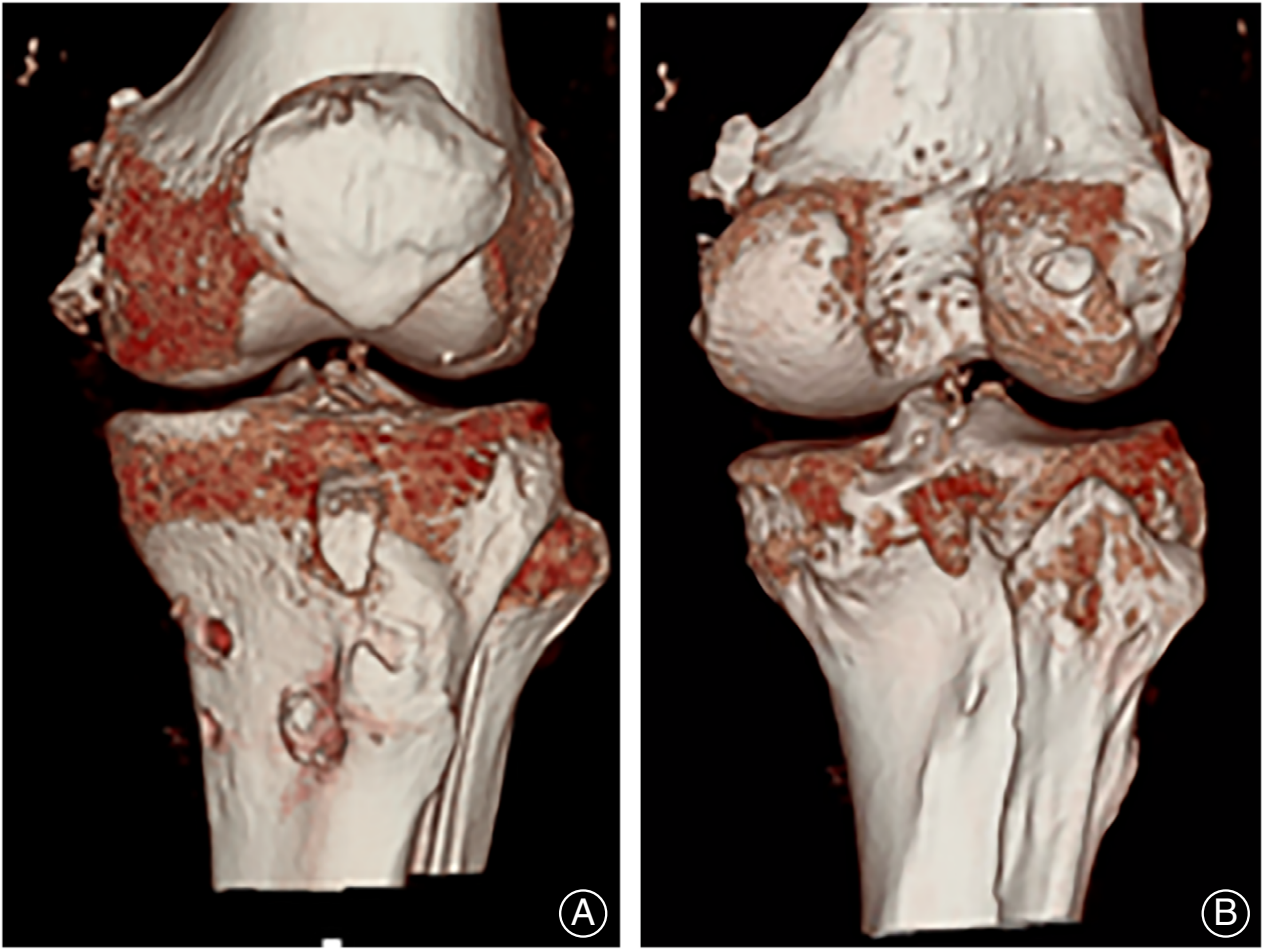
Postoperative conditions. (A, B) 3D reconstruction of the reconstructed knee

**Fig. 5:**
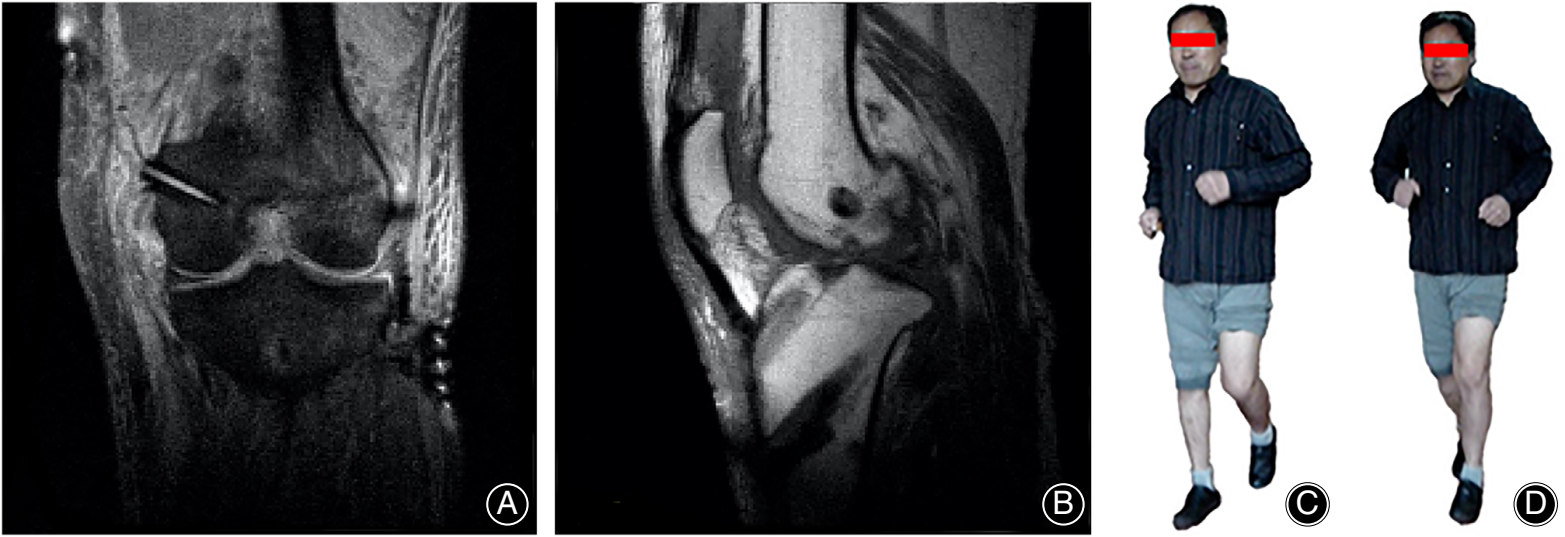
Short‐term follow‐up data. (A, B) Anteroposterior and lateral MRI views of the knee at the 3rd month postoperatively. (C, D) The patient could participate in moderate‐intensity sports 4 months postoperatively

As an illustrative example, we present the individual data of one patient who received treatment for Schenck IV dislocation of the left knee in 2010 and was followed up for more than 10 years. This patient's preoperative, intraoperative, postoperative conditions and follow‐up data are shown in Figures 2, 3, 4, 5 and Video S1.

## Discussion

**Fig. 6:**
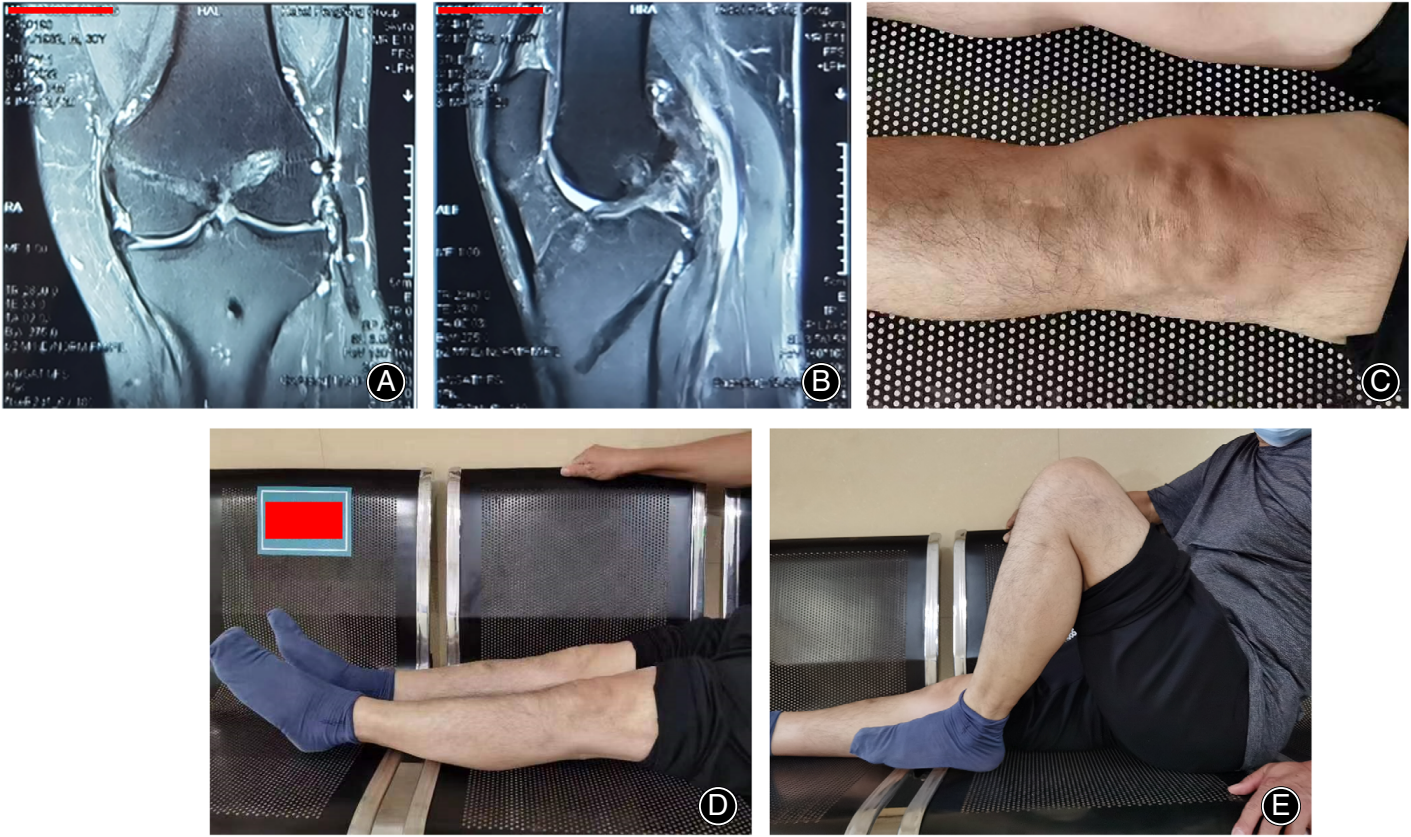
Long‐term follow‐up data. (A, B) Anteroposterior and lateral MRI views of the knee at the 12th year postoperatively. (C) Appearance of incisions 12 years postoperatively. (D, E) The ROM at the 12‐year postoperative follow‐up was satisfactory

In the present study, we have illustrated a new one‐stage arthroscopic multiple ligament reconstruction technique which shows its effectiveness in treating Schenck IV KD with satisfactory long‐term knee function and relatively low complications. This surgical technique provided us with a new option facing Schenck IV KD patients (Figure 6).

### Current Treatment Options

The traditional treatments of multiligament‐injured KD (MLIKD) include conservative treatment,^12^, ^13^ *in situ* suture surgery,^14^, ^15^, ^16^ and ligament reconstruction surgery.^17^ Although patients who receive operative treatment show better knee function than patients who do not receive operative treatment,^8^, ^10^, ^18^, ^19^ it is still unknown which surgical method is best for treating MLIKD. Some studies have shown satisfactory results when using the ligament reconstruction technique to treat MLIKD patients,^19^, ^20^, ^21^, ^22^, ^23^ while others have shown that *in situ* ligament repair is more effective.^24^, ^25^, ^26^, ^27^, ^28^ Due to the heightened knee instability that is characteristic of Schenck IV KD, we chose ligament reconstruction as our treatment option, and the patients were encouraged to follow a strict rehabilitation program. This treatment plan demonstrated satisfactory results in treating Schenck IV KD patients.

### Advantages of this Technique

The arthroscopic one‐stage multiligament reconstruction technique has several advantages in treating Schenck IV KD patients. First, these patients need only a one‐stage procedure, which allows them to avoid the mental, physical, and financial burden of multiple‐stage operations. Second, this method provides instant knee stability and allows the patient to begin a strict knee rehabilitation regimen at a relatively early stage to avoid knee stiffness, which has been recognized as one of the major complications of MLIKD.^10^ In our study, the patients treated with the one‐stage multiligament reconstruction technique showed satisfactory knee function at the last follow‐up, and the rate of knee stiffness was lower in this study than in most studies focusing on the treatment of KD.^4^, ^15^, ^29^ Third, the operation time is shorter than that of other ligament reconstruction treatments. Single‐stage multiligament reconstruction demonstrates even more benefits in cases of severely damaged tissues, with reduced operative times and consequentially decreased risks of time‐related intraoperative complications. Finally, a frozen homologous Achilles tendon provides enough strength without disturbing the autologous tendon and offers greater knee stability than an autogenous tendon; it also saves time by averting the need to harvest an autologous tendon. This surgical method is our own novel design and follows the principles of isometric reconstruction. To our knowledge, no similar method has been performed in other studies. A recent study by Dustin et al.^4^ presented five patients treated according to individual plans, and four ligament reconstruction surgeries were eventually conducted; all five patients showed satisfactory knee function.

### Optimal Time for Surgery

The optimal time for operation is still controversial. The surgeries were divided into early‐stage surgery and delayed surgery using a cutoff of 3 weeks after trauma. Some cohort studies have shown that early‐stage surgery yields better Lysholm and IKDC scores than delayed surgery,^28^, ^30^, ^31^, ^32^, ^33^ while some studies have reported that the incidence of knee stiffness is higher in patients who received early‐stage surgery.^10^, ^34^ Hantes et al.^21^ reported a case series containing 26 TKD patients who received delayed reconstruction surgery with a mean follow‐up time of 105 months. There was no statistical significance in the range of motion compared to normal, uninjured people. However, that study did not include patients with Schenck IV or V KD. Mook et al.^35^ reviewed studies of severe multiligament knee injury (KD III and higher) in 2009; they concluded that early surgeries resulted in significantly worse range of motion deficits and a need for additional treatments for joint stiffness and that there were no differences in Lysholm scores between patients treated early and patients treated on a delayed basis. Jiang et al.^36^ published a systematic review evaluating surgical timing among KD III patients, and no difference was found between the early and delayed surgical groups. In a systemic review, Hohmann et al.^37^ noted significantly increased Lysholm scores among patients who underwent early surgery while discovering no difference in range of motion between the early and delayed groups. A recent systematic review by Sheth et al.^29^ analyzed 11 eligible studies that included a total of 320 patients (195 early and 125 delayed) and concluded that early surgery led to statistically significantly higher Lysholm scores (*p* < .0001) and Meyers ratings (*p* = .02) than delayed surgery.

We suggest performing surgery in a time window of 0–3 weeks post‐injury. Emergency operation is needed if there is serious swelling, combined irreducible KD, joint capsule incarceration, or compartment syndrome. In our study, patients who underwent surgery within 3 weeks of injury had better knee function than those who underwent surgery after 3 weeks. We suggest that this time window (0–3 weeks) may have several benefits. First, early surgery provides instant knee stability and creates a recuperative environment that promotes the healing of other knee structures, such as the posterolateral corner. Second, it avoids complications related to serious swelling. Third, ligament reconstruction surgery allows the patient to undergo early rehabilitation, which is very important to avoid postoperative knee stiffness. Last, it reduces the knee immobilization time and the suffering and medical expenses borne by the patient. This surgical intervention period was also supported by Fanelli in a recent review of TKD.^38^

Experienced senior surgeon in the sports field is important. First, their experience can help reduce the tourniquet time. A prolonged tourniquet time could exacerbate tissue ischemia and propagate thrombus formation in the setting of an intimal tear. Additionally, saline extravasation from the injured joint capsule may cause swelling of the knee joint and the lower limb, thus increasing the difficulty of the operation and sometimes affecting the tissue healing or even causing compartment syndrome.^39^, ^40^ The bone tunnels were precisely designed to ensure that there was no tunnel convergence. However, a slight shift in the tunnel may cause tunnel convergence. The surgeon's experience will be helpful in avoiding tunnel convergence and adjusting the directions accordingly, thus reducing intraoperative complications.

### Strengths and Limitations

Our study has its strength, the surgical plan was carefully planned based on the anatomical structure of the knee. The follow‐up data of the patient group show its effectiveness with satisfactory knee function result. The limitations of our study may be the retrospective nature and relatively low number of cases. However, the low morbidity of Schenck IV KD makes it especially difficult to perform a prospective randomized controlled study.^41^ To our knowledge, our study is the largest case series focusing on Schenck IV KD patients. Another limitation of this study is that we did not have cases with serious artery or nerve injuries. This may be reasonable, since we seldom performed this surgery on Schenck IV KD patients with artery injuries (which is relatively dangerous) or nerve injuries (which will affect the postoperative rehabilitation process). A multicenter study with a larger number of cases is needed to further evaluate this new arthroscopic one‐stage multiligament reconstruction technique for the treatment of Schenck IV KD patients.

### Conclusions

This new one‐stage frozen homologous Achilles tendon multiligament reconstruction technique is effective in treating patients with Schenck IV KD. Patients treated with this method achieved satisfactory knee function. A prospective, multicenter, randomized controlled study is needed for further evaluation.

## Supporting information


**Video S1.** Multimedia data from the 12‐year postoperative follow‐up show a good long‐term prognosis

## References

[1] Dhariwal H , Tholgapiyan T , Ashokan C , et al. Successful management of a bilateral knee fracture dislocation with multi‐ligament insufficiency sustained while getting off a moving train—a rare case report. J Orthop Rep. 2022;1:33–7.

[2] Smith J‐RH , Belk JW , Friedman JL , et al. Predictors of mid‐ to long‐term outcomes in patients experiencing a knee dislocation: a systematic review of clinical studies. J Knee Surgery. 2022;35(12):1333–1341. 33545729 10.1055/s-0041-1723762

[3] Boyce RH , Singh K , Obremskey WT . Acute management of traumatic knee dislocations for the generalist. J Am Acad Orthop Surg. 2015;23(12):761–8. 26493970 10.5435/JAAOS-D-14-00349

[4] Richter DL , Bankhead CP , Wascher DC , Treme GP , Veitch A , Schenck RC Jr . Knee dislocation (KD) IV injuries of the knee: presentation, treatment, and outcomes. Clin Sports Med. 2019;38(2):247–60. 30878047 10.1016/j.csm.2018.11.007

[5] Azar FM , Brandt JC , Miller RH III , Phillips BB . Ultra‐low‐velocity knee dislocations. Am J Sports Med. 2011;39(10):2170–4. 21757779 10.1177/0363546511414855

[6] Rosteius T , Jettkant B , Rausch V , Lotzien S , Königshausen M , Schildhauer TA , et al. Anatomical repair and ligament bracing of Schenck III and IV knee joint dislocations leads to acceptable subjective and kinematic outcomes. Knee Surg Sports Traumatol Arthrosc. 2021;29:4188–97. 33688978 10.1007/s00167-021-06501-2 PMC8595154

[7] Schenck JR . The dislocated knee. Instr Course Lect. 1994;43:127–36. 9097143

[8] Everhart JS , Du A , Chalasani R , et al. Return to work or sport after multiligament knee injury: a systematic review of 21 studies and 524 patients. Arthroscopy. 2018;34(5):1708–16. 29429563 10.1016/j.arthro.2017.12.025

[9] Werner BC , Hadeed MM , Gwathmey FW , et al. Medial injury in knee dislocations: what are the common injury patterns and surgical outcomes? Clin Orthop Relat Res. 2014;472(9):2658–66. 24500780 10.1007/s11999-014-3483-3 PMC4117899

[10] Cook S , Ridley T , McCarthy MA , et al. Surgical treatment of multiligament knee injuries. Knee Surg Sports Traumatol Arthrosc. 2015;23(10):2983–91. 25427976 10.1007/s00167-014-3451-1

[11] Li T , Xiong Y , Zhang Z , Tang X , Chen G , Li Q , et al. Results of multiple ligament reconstruction after knee dislocation——a prospective study with 95 patients and minimum 2‐year follow up. BMC Musculoskelet Disord. 2021;22(1):1–13. 34706679 10.1186/s12891-021-04596-9 PMC8554847

[12] Peskun CJ , Whelan DB . Outcomes of operative and nonoperative treatment of multiligament knee injuries: an evidence‐based review. Sports Med Arthrosc Rev. 2011;19(2):167–73. 21540715 10.1097/JSA.0b013e3182107d5f

[13] Venter S‐M , Dey R , Khanduja V , et al. The management of acute knee dislocations: a global survey of orthopaedic surgeons' strategies. SICOT‐J. 2021;7:21. 33812447 10.1051/sicotj/2021017 PMC8019554

[14] Jonkergouw A , van der List JP , DiFelice GS . Multiligament repair with suture augmentation in a knee dislocation with medial‐sided injury. Arthrosc Tech. 2018;7(8):e839–43. 30167362 10.1016/j.eats.2018.04.006 PMC6112193

[15] Heitmann M , Akoto R , Krause M , et al. Management of acute knee dislocations: anatomic repair and ligament bracing as a new treatment option—results of a multicentre study. Knee Surg Sports Traumatol Arthrosc. 2019;27(8):2710–2718. 30631909 10.1007/s00167-018-5317-4

[16] Murakami R , Honda E , Fukai A , et al. Single‐stage arthroscopic anterior and posterior cruciate ligament repairs and open medial collateral ligament repair for acute knee dislocation. Case Rep Orthopedics. 2020;2020:7348201. 10.1155/2020/7348201 PMC7060436 32158579

[17] Khakha R , Day A , Gibbs J , et al. Acute surgical management of traumatic knee dislocations—average follow‐up of 10 years. Knee. 2016;23(2):267–75. 26545616 10.1016/j.knee.2015.09.019

[18] Li T , Xiong Y , Zhang Z , Tang X , Chen G , Li Q , et al. Results of multiple ligament reconstruction after knee dislocation——a prospective study with 95 patients and minimum 2‐year follow up. BMC Musculoskelet Disord. 2021;22:904. 34706679 10.1186/s12891-021-04596-9 PMC8554847

[19] Xing X , Shi H , Feng S . Does surgical treatment produce better outcomes than conservative treatment for acute primary patellar dislocations? A meta‐analysis of 10 randomized controlled trials. J Orthop Surg Res. 2020;15:118. 32209111 10.1186/s13018-020-01634-5 PMC7093955

[20] Tao J , Li X , Zhou Z , Zhu Z . Acute single‐stage reconstruction of multiligament knee injuries using the ligament advanced reinforcement system. Med Princ Pract. 2013;22(4):373–8. 23428973 10.1159/000346663 PMC5586766

[21] Hantes M , Fyllos A , Papageorgiou F , Alexiou K , Antoniou I . Long‐term clinical and radiological outcomes after multiligament knee injury using a delayed ligament reconstruction approach: a single‐center experience. Knee. 2019;26(6):1271–7. 31575512 10.1016/j.knee.2019.08.009

[22] King AH , Krych AJ , Prince MR , Pareek A , Stuart MJ , Levy BA . Surgical outcomes of medial versus lateral multiligament‐injured, dislocated knees. Arthroscopy. 2016;32(9):1814–9. 27062009 10.1016/j.arthro.2016.01.038

[23] Suh JT , Ahn JM , Lee JM , Kim NR . Staged surgical management of multiple ligament injury of the knee. Arthrosc Orthop Sports Med. 2014;1(2):111–9.

[24] Hua X , Tao H , Fang W , Tang J . Single‐stage in situ suture repair of multiple‐ligament knee injury: a retrospective study of 17 patients (18 knees). BMC Musculoskelet Disord. 2016;17(1):41. 26801911 10.1186/s12891-016-0894-1 PMC4722785

[25] Owens BD , Neault M , Benson E , Busconi BD . Primary repair of knee dislocations: results in 25 patients (28 knees) at a mean follow‐up of four years. J Orthop Trauma. 2007;21(2):92–6. 17304061 10.1097/BOT.0b013e3180321318

[26] Hanley JM , Anthony CA , DeMik D , Glass N , Amendola A , Wolf BR , et al. Patient‐reported outcomes after multiligament knee injury: MCL repair versus reconstruction. Orthop J Sports Med. 2017;5(3):2325967117694818. 28357408 10.1177/2325967117694818 PMC5358815

[27] Heitmann M , Akoto R , Krause M , Hepp P , Schöpp C , Gensior TJ , et al. Management of acute knee dislocations: anatomic repair and ligament bracing as a new treatment option—results of a multicentre study. Knee Surg Sports Traumatol Arthrosc. 2018;27:2710–8. 10.1007/s00167-018-5317-4 30631909

[28] Vermeijden HD , Jonkergouw A , van der List JP , DiFelice GS . The multiple ligament‐injured knee: when is primary repair an option? Knee. 2020;27:173–82. 31926671 10.1016/j.knee.2019.11.013

[29] Sheth U , Sniderman J , Whelan DB . Early surgery of multiligament knee injuries may yield better results than delayed surgery: a systematic review. J ISAKOS. 2019;4(1):26–32.

[30] Richter M , Bosch U , Wippermann B , Hofmann A , Krettek C . Comparison of surgical repair or reconstruction of the cruciate ligaments versus nonsurgical treatment in patients with traumatic knee dislocations. Am J Sports Med. 2002;30(5):718–27. 12239009 10.1177/03635465020300051601

[31] Ríos A , Villa A , Fahandezh H , et al. Results after treatment of traumatic knee dislocations: a report of 26 cases. J Trauma Acute Care Surg. 2003;55(3):489–94. 10.1097/01.TA.0000043921.09208.76 14501892

[32] Wong C‐H , Tan J‐L , Chang H‐C , Khin LW , Low CO . Knee dislocations—a retrospective study comparing operative versus closed immobilization treatment outcomes. Knee Surg Sports Traumatol Arthrosc. 2004;12(6):540–4. 14999440 10.1007/s00167-003-0490-4

[33] Tardy N , Boisrenoult P , Teissier P , Steltzlen C , Beaufils P , Pujol N . Clinical outcomes after multiligament injured knees: medial versus lateral reconstructions. Knee Surg Sports Traumatol Arthrosc. 2017;25(2):524–31. 27000392 10.1007/s00167-016-4067-4

[34] Harner CD , Waltrip RL , Bennett CH , Francis KA , Cole B , Irrgang JJ . Surgical management of knee dislocations. J Bone Jt Surg. 2004;86(2):262–73. 10.2106/00004623-200402000-00008 14960670

[35] Mook WR , Miller MD , Diduch DR , Hertel J , Boachie‐Adjei Y , Hart JM . Multiple‐ligament knee injuries: a systematic review of the timing of operative intervention and postoperative rehabilitation. J Bone Jt Surg. 2009;91(12):2946–57. 10.2106/JBJS.H.01328 19952260

[36] Jiang W , Yao J , He Y , Sun W , Huang Y , Kong D . The timing of surgical treatment of knee dislocations: a systematic review. Knee Surg Sports Traumatol Arthrosc. 2015;23(10):3108–13. 25408556 10.1007/s00167-014-3435-1

[37] Hohmann E , Glatt V , Tetsworth K . Early or delayed reconstruction in multi‐ligament knee injuries: a systematic review and meta‐analysis. Knee. 2017;24(5):909–16. 28716470 10.1016/j.knee.2017.06.011

[38] Fanelli GC . Knee dislocation and multiple ligament injuries of the knee. Sports Med Arthrosc Rev. 2018;26(4):150–2. 30395055 10.1097/JSA.0000000000000220

[39] Tay AK , MacDonald PB . Complications associated with treatment of multiple ligament injured (dislocated) knee. Sports Med Arthrosc Rev. 2011;19(2):153–61. 21540713 10.1097/JSA.0b013e31820e6e43

[40] Chowdhry M , Burchette D , Whelan D , Nathens A , Marks P , Wasserstein D . Knee dislocation and associated injuries: an analysis of the American College of Surgeons National Trauma Data Bank. Knee Surg Sports Traumatol Arthrosc. 2019;28:568–75. 31559462 10.1007/s00167-019-05712-y

[41] Hankins DA , Fletcher IE , Prieto F , Ockuly AC , Myers OB , Treme GP , et al. Critical evaluation of the methodologic quality of the top 50 cited articles relating to knee dislocation and multiligamentous knee injury. Orthop J Sports Med. 2019;7(11):2325967119880505. 31742213 10.1177/2325967119880505 PMC6843738

